# Notes and News

**Published:** 1899-10-14

**Authors:** 


### Notes and News.

(Collected and Communicated.)

The Chelsea Hospital for Women is added to the number of recently reopened hospitals. The operating theatre has been enlarged and improved, the electric light has been installed, and a new hot-water service and electric lift have been added.

Professor Haffkine was a guest at the recently-held annual dinner of old students of the London Hospital. In responding to the toast of "The Guests," he spoke a few words about the plague, but stated that all his researches had impressed him with the fact that there was much to learn.

The Public Health Congress held at Blackpool, from September 21st to 28th, was opened by the Marquis of Lorne, and was a success. Many useful papers were read, and discussions took place. A large number of medical officers of health took part in the proceedings, and the Chief Rabbi's paper on the sanitation of the Mosaic law created special interest.

We note with interest, not altogether unmixed with amusement, that the *Australasian Medical Gazette*, which is the journal of the Australasian branches of the British Medical Association, has inserted a letter from Dr. W. Ramsay Smith, senior physician to the Adelaide Hospital, on the treatment of diphtheria, in which he deals very severely with the president of the South Australian branch. Time tempers all things, and after seeing the Australian branches make the British Medical Association dance to their own tune a year or two ago, there is a certain oddity in finding their own journal (which, by-the-bye, is not published in Adelaide) admitting to its columns a letter from one of the gentlemen about whose action in accepting a Government appointment such a fuss was then made, a letter in which he is allowed not only to deal with the question of diphtheria, but also to give the president of the South Australian branch a very good wigging.

The plague is not yet stamped out at Oporto. Fresh cases continue to show themselves, and ignorance and prejudice render the work of the doctors much harder.

Major Ross and other members of the Liverpool malaria expedition arrived at Plymouth last Monday on their return from the West Coast of Africa. He stated that although the expedition had only been at work for six weeks, it had been very successful, and the authorities at Sierra Leone had determined to use every means to exterminate the malaria-spreading mosquito. Putting malaria on one side, other conditions of life in West Africa were favourable to health; but he thought the whites were not careful enough, and that their houses were badly constructed, and compared very unfavourably with the residences of whites in India, which were so built as to give to their inhabitants every chance of health, despite the climate. One member of the expedition, Mr. Austin, had, he said, suffered from malaria. He slept for one night without mosquito curtains, and was thus infected. He was, however, now convalescent.

In a report of the proceedings of the Lambeth County Court we see that it is stated that, on a medical certificate being handed up in support of an application to adjourn a case, Judge Emden said he would never again pay any attention to medical certificates which did not specifically state the complaint the patient suffered from. The doctor, in this instance, wrote: "I consider him unfit to attend the Court." That was not a question for a medical man to decide—it was for the Court to decide. Now, we have a great respect for Judge Emden. On many questions he has shown a degree of common sense and adaptability not very common in members of the legal profession. On this particular matter, however, he seems to have made a slip. We should like to see "the Court" endeavouring to make up its mind whether a man was fit or not to attend, if the doctor had strictly limited his certificate to a specific statement of the complaint from which his patient was suffering. The fact is, that the statement of the fitness of the man to attend the Court is the very essence of the certificate.

No branch of the services is moved about so much as the officers of the Royal Army Medical Corps, and the cost of providing themselves with the various kinds of equipment required in different parts of the world to which they may be sent is no small item in their annual expenses. It would seem, then, by no means an unreasonable concession that they should be allowed to purchase at cost price all such things as can be supplied from the Government stores, even to khaki uniform, instead of having to pay the high prices charged for them by the outfitters. The constant moves to which these officers are exposed entail far greater expense upon them than upon regimental officers; and all this, which is a loss to them, is no gain to the country. The question of horses in time of active service is also a serious affair; but, in regard to that, we believe officers going to South Africa will be provided with mounts at cost price by the Government, which is something. What they will get for the said horses when the expedition returns does not, however, seem very clear. Probably but little.

